# Intelligent Congestion Control in the Internet of Vehicles Employing Network Slicing in 5G Architecture

**DOI:** 10.3390/s26134154

**Published:** 2026-07-01

**Authors:** Arbab Waheed Ahmad, Raja Sana Gul, Mohammad Derawi

**Affiliations:** 1 Department of Electrical and Computer Engineering, Pak-Austria Fachhochschule Institute of Applied Sciences and Technology, Haripur 22621, Pakistan; waheed.arbab@fecid.paf-iast.edu.pk; 2 Department of Electronic Engineering, Maynooth University, W23 F2H6 Maynooth, Ireland; raja.gul.2025@mumail.ie; 3 Department of Electronic Systems, Faculty of Information Technology and Electrical Engineering, Norwegian University of Science and Technology, 7034 Trondheim, Norway

**Keywords:** resource allocation, internet of vehicles, network slicing, software-defined networking, V2X communication

## Abstract

The Internet of Vehicles (IoV) is a decentralized network architecture that enables autonomous driving, real-time applications, infotainment services, and seamless vehicle-to-everything communication. While infotainment systems enhance the user experience by providing entertainment and navigation features, their high data demands can cause network congestion, potentially delaying mission-critical messages and compromising safety and reliability. Further, the increasing volume of connected devices and data traffic exacerbates these challenges, resulting in high latency, low throughput, and reduced network efficiency. To address this, we propose a novel lightweight adaptive network slicing strategy with four dedicated slices and priority-weighted dynamic bandwidth allocation for IoV networks to mitigate congestion and ensure the availability of the required bandwidth for critical communications. The proposed mechanism defines four distinct network slices, allocating resources to balance infotainment and mission-critical needs. Simulation results demonstrate that our approach achieves ultra-low latency (<5 ms), near-zero packet loss (<0.5%), and high throughput (53 Mbps for infotainment), significantly outperforming existing methods, and ensuring reliable communication for safety-critical tasks while improving spectrum utilization. Findings validate implementing network slicing in IoV environments, paving the way for efficient, congestion-free, and high-performance vehicular networks.

## 1. Introduction

The telecommunications industry has undergone a significant transformation in recent years, moving away from traditional wired telephone services to data-centric services. In addition, traditional devices have been replaced by portable smart mobile computers [1,2]. In recent years, the number of handheld devices has increased along with the demand for high-speed information applications. To meet the requirements of next-generation applications, existing data rates need to be improved [3]. There has been a significant increase in the use of cellular networks for data delivery. This trend is expected to continue due to the widespread use of smartphones and the growing number of services designed for them. To keep up with the demands of emerging services, cellular networks have been continuously evolving. For example, 2G networks provided the first digital infrastructure for data services such as SMS, 3G enabled mobile internet usage and video calls, and 4G paved the way for gaming and video conferencing services over cellular networks [4].

The increasing popularity of mobile video services, including YouTube, Mobile TV, and advances in Internet of Things (IoT) technology, has driven the ongoing global deployment and expansion of 5G mobile and wireless communication systems. The growing number of smart devices and bandwidth-intensive mobile applications pose significant challenges to current 5G networks in terms of spectral efficiency. The Cisco Visual Networking Index (VNI) Forecast estimates that in the coming years, IP video traffic will make up 82% of all consumer Internet traffic and 78% of global mobile data traffic. To meet the demands of this connected society, 5G network systems provide significantly more capacity than 4G systems. 5G improves coverage, increases user data rates, saves energy, and reduces latency. Additionally, it is projected that by 2035, 5G will contribute to a global economic output of $12.3 trillion [5].

5G represents mobile communication networks designed to provide ultra-high-speed, ultra-reliable, and ultra-low-latency communication to users. This can be achieved by incorporating advanced technologies such as ultra-dense heterogeneous network deployment [6], device-to-device (D2D) communication [7], cognitive radio [5], multiple-input multiple-output (MIMO) in large-scale networks, and more. The 5G network holds the potential to revolutionize various industries, including, but not limited to cars, healthcare, energy, media, and entertainment [8,9].

**Figure 1:**
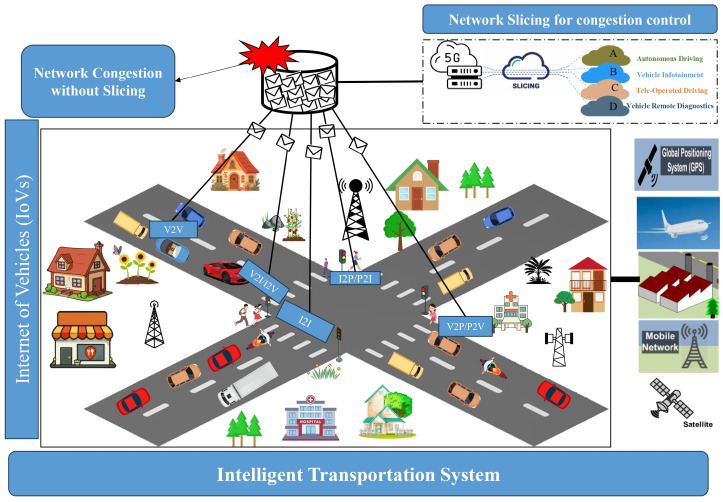
Proposed Network Slicing Framework.

5G introduces network slicing (NS), which allows network resources to be divided into multiple virtual networks. Each network slice can be customized to meet specific requirements, such as bandwidth, latency, and security, to support various applications. Network slicing enables efficient resource allocation, ensuring that different services receive the network performance they need. The benefits of network slicing and reconfiguration include reduced latency, high bandwidth, seamless mobility, and improved QoS [10]. 5G also allows network operators to dynamically allocate resources, adapt to changing demands, and introduce new services more efficiently in various fields. One significant field is the IoV, a decentralized network paradigm designed to enable secure, seamless value transfer across various blockchain networks as the number of vehicles increases rapidly each year [11]. The rapidly developing field of the IoV has a wide range of applications, including intelligent traffic management, wireless audience monitoring, hazard identification, weather warnings, surveillance, parking warning lights, and in-car streaming media. The IoV also highlights essential locations along a route, such as fuel stations, parking lots, and shops. Additionally, it can be used in E-health to provide mobile hospital services. The IoV aims to ensure road safety, regulate traffic through signals, and quickly report accidents through information exchange [12]. The vehicle communication system encompasses various components, including vehicles, cloud networks, road units, the internet, fog networks, people, and pedestrian transportation devices. The primary goal of this system is to improve road safety, reduce fuel consumption and CO2 emissions, save time, and provide a new driving experience. The type of communication is called “V2X contact” and encompasses various forms of communication between vehicles, such as V2V, V2I, V2N, and V2P. V2X communication plays a crucial role in the development of Connected Vehicle (CV) technology, enabling vehicles to interact with individuals and entities in their surroundings [13,14]. Vehicles typically communicate in two ways: (1) regular updates (beacons), where they share information including position, speed, and yaw angle with each other; and (2) emergency alerts, where they send out messages if there is a crisis, such as an accident or a fire. Suppose too many vehicles send beacons frequently, or there are too many emergency alerts—the communication channel can become congested, blocking some important emergency messages from reaching their destinations and compromising safety [15]. The IoV faces a central issue of congestion during information dissemination. Existing schemes handle this issue through congestion detection and alternate path selection. Most of these schemes are designed for Vehicle Ad hoc Networks (VANETs) [8,16]. The proposed research focuses on adopting a network slicing-based strategy to mitigate network congestion in IoV networks for a congestion-free network with low latency, high throughput, and effective use of network resources based on network slicing, as presented in Figure 1.

The main contributions of this paper are:

- This paper proposes a lightweight adaptive network slicing mechanism for IoV that dynamically reallocates bandwidth between four dedicated slices using priority-based weights, achieving ultra-low latency and near-zero packet loss for URLLC slices without requiring heavy ML training or edge computing dependencies.
- We implement a priority-weighted dynamic bandwidth allocation, enabling real-time adaptability in fast-moving vehicular environments while ensuring performance isolation between mission-critical V2V/teleoperation traffic and best-effort infotainment flows.
- Through extensive NS-3.37/5G-LENA-v2.3 simulations, we evaluate the proposed mechanism across throughput, delay, and packet loss ratio metrics under varying vehicle densities. The results demonstrate significant improvements over existing approaches with Slice B achieving 53 Mbps throughput and Slices A, C, D maintaining <5 ms delay and <0.5% packet loss at 200 nodes.

The rest of the paper is organized as follows. Section 2 reviews previous strategies. Section 3 discusses the proposed methodology adopted in designing end-to-end congestion control for cellular networks. Then, Section 4 presents and discusses the simulation results. Conclusions are drawn in Section 5.

## 2. Related Work

Early research in vehicular networks primarily focused on traditional congestion control mechanisms that employed various algorithmic approaches to manage network traffic. These methods typically involve power control, rate adaptation, or utility-based forwarding to mitigate congestion in VANETs. Ref. [12] proposes a congestion-avoidance technique for VANETs using the energy-efficient message dissemination (E2MD) scheme, which involves continuous updating of vehicle information to a fog server. The authors also address the limitation of fog server failures by proposing an algorithm for server failure detection and recovery. One limitation of this work is the high delay in accessing resources and responding to requests, which can impact the overall system performance. In [17], the authors propose a congestion control scheme for NR-V2X sidelink that uniquely maximizes effective throughput by jointly optimizing the modulation and coding scheme, resource block allocation, and message period. Using a stochastic geometry model and the NSGA-II algorithm, the scheme finds Pareto-optimal settings, demonstrating significant throughput gains (5.48% to 863.69%) while maintaining Channel Busy Ratio below a congestion threshold.

The authors in [18] present an Echelon-Based Collaborative Resource Allocation (ECRA) protocol to address packet collisions in C-V2X Mode 4 platooning. This approach reduces packet collision probability by 91.8% and average delay by 15.3% in simulations. A key limitation is the increased algorithmic complexity from the fuzzy logic and multi-mechanism design, which may burden resource-constrained onboard units, and its performance in very large or extreme scenarios requires further validation. In [19], the author introduces an intelligent traffic congestion detection system that integrates cognitive intelligence with VANETs. The method uses Fuzzy K-Means clustering and the Fuzzy Analytical Hierarchy Process (FAHP) to analyze real-time vehicle data from a SUMO simulation, effectively identifying congestion hotspots. The primary limitations are its reliance on simulated data, which may not reflect real-world complexities, and susceptibility to VANET challenges like communication delays and link breakages in dense urban environments.

With the advent of 5G and beyond, network slicing has emerged as a promising paradigm for resource management in the IoV. This approach creates virtualized logical networks on a shared physical infrastructure, allowing customized performance characteristics for different service types. Recent works have explored various slicing architectures and allocation strategies specifically for vehicular environments. In [20], the authors use a strategic approach based on software-defined multiple access (SoDeMa) algorithms to enhance network traffic performance. The proposed network slicing algorithm design reduces complexity and improves reaction time. The proposed framework in [21] utilizes slice management and an SDN controller to coordinate network resources and achieve the priorities and Key Performance Indicators (KPIs) of Intelligent Transportation System (ITS) applications. The slicing mechanism assumes stable network environments, which may not hold in highly dynamic vehicular scenarios where latency and handover are critical. In [22], a mechanism for establishing and managing network slices in Wi-Fi RANs for 5G is proposed. They introduce a slicing approach compatible with the IEEE 802.11 standard [23] and algorithms for slice creation and resource management based on 5G QoS Identifiers. The authors in [24] presents a network slicing architecture for the IoV based on Multi-Access Edge Computing (MEC). The architecture includes Domain Orchestrators (DOs) and a General Orchestrator (GO) to provide reliable, differentiated services. The evaluation is limited to a small-scale prototype and does not demonstrate scalability to large city-wide vehicular systems, and, most importantly, a heavy reliance on edge computing can raise costs and management overhead in real deployments. In [25], a programmable, dynamic end-to-end slicing mechanism is proposed for an M-CORD-based LTE network. The solution utilizes the Network Slice Selection Function (NSSF) and open-source components on the M-CORD platform. The architecture has not been validated in live vehicular settings, making it unclear how well it performs under fast handovers or congestion. In [26], the author proposes a solution for resource allocation in Wi-Fi Access Points (APs) for network slices in 5G networks. Their solution is based on queuing and scheduling policies to allocate time slices while ensuring isolation and performance requirements. The limitation is that it offers no guarantees on throughput or latency, which are critical for vehicular safety applications.

The authors in [27] introduce the concept of network slicing as a service (NSaaS) and provide a technical solution for its implementation. The solution combines the benefits of network functions virtualization (NFV), software-defined networks (SDN), and control plane solutions, but lacks the real simulation results. In [28], an end-to-end (E2E) network slicing methodology is proposed that considers both radio access network (RAN) and core network (CN) resources. Simulations using the Genetic Algorithm show the effectiveness of the framework. A limitation is that Genetic Algorithms can be slow in real-time decision-making, posing a risk in highly mobile, latency-sensitive VANETs. Ref. [29] discusses network slicing design for vehicle-to-everything services in 5G networks. The authors emphasize the importance of Network Function Virtualization (NFV), Software Defined Networks (SDN), and Mobile Edge Computing (MEC) for ITS. The authors do not demonstrate how their slicing model affects key QoS parameters, such as latency, packet loss, or service availability, in a real or simulated V2X context. Ref. [30] proposes a smart slice scheduling approach in vehicle fog radio access networks to handle complex and dynamic network traffic in the IoV. The approach utilizes a Markov decision process and a clever algorithm for efficient resource allocation, which is limited to a small scale and a controlled environment. There is no consideration of dense areas or congestion, which is critical for real-world vehicular slicing deployments.

Recent advancements in artificial intelligence have inspired numerous learning-based approaches for congestion control and resource management in the IoV. These methods leverage machine learning, deep learning, and reinforcement learning to optimize network performance through data-driven decision making. While [31] discusses AI-based methods, including reinforcement and deep learning, for controlling vehicle networks via 5G in the context of the IoV. To allocate radio resources in a 5G IoV network, this paper examined various algorithms but lacked real simulation results. The authors in [32] propose hybrid deep learning approaches for intelligent traffic congestion control in 5G/6G networks. They develop a generic architecture for congestion detection using a hybrid deep learning algorithm. Ref. [33] addresses resource allocation in IoV networks facilitated by MEC. The authors suggest a model-free method based on deep reinforcement learning (DRL) and non-orthogonal multiple access (NOMA) to improve resource utilization, which requires significant training time and data, making real-time deployment and adaptation challenging in fast-moving vehicular networks. In [34], the authors present an ML-driven Automatic Network Slicing (ANS) framework for smart cities. The framework includes online ML-driven prediction, unsupervised learning-based anomaly detection, and reinforcement learning-based scaling for network slices, but lacks real-world urban traffic scenarios.

The authors of [35] propose an adaptive scheduling scheme that jointly optimizes road traffic flow and IoV network resources using Lyapunov-assisted Q-learning and RCDT algorithms to maximize throughput. Their approach shows 19.92% higher throughput but assumes full task offloading and faces computational complexity in large-scale networks, while our work uses simpler priority-based slicing without offloading dependencies. Ref. [36] develops an LSTM-based proactive congestion management framework achieving 98% prediction accuracy with packet prioritization via K-means clustering. However, their centralized approach and simulated dataset limit scalability and real-world applicability, whereas our distributed slicing architecture performs real-time validation under mobility. Ref. [37] presents a resource-aware cooperative traffic control scheme using multi-agent reinforcement learning with attention mechanisms for intersection cooperation. While effective for throughput, their method relies on strong vehicle-following behavior and faces scalability issues, unlike our slice isolation, which guarantees performance without behavioral assumptions. Ref. [8] surveys ML-based network slicing for IoV, highlighting advantages in QoS differentiation and slice isolation but noting computational demands and security vulnerabilities of ML models. Their work covers NS architecture, enabling technologies (SDN/NFV/MEC), slicing methods (static, semi-dynamic, dynamic), and the integration of machine learning techniques for network slicing in the IoV. The authors review NS-based applications across terrestrial, aerial, and maritime domains, along with V2X datasets, simulators, and relevant projects. Our framework avoids complex ML training through lightweight dynamic allocation, ensuring real-time operation in high-mobility scenarios. An emerging direction in this domain is the concept of Distributed Learning as a Service (DLaaS) over network slicing, as recently proposed by [38] for 6G non-terrestrial network (NTN) scenarios. Their framework integrates distributed learning methods with network slicing to enable intelligent service delivery across integrated terrestrial and non-terrestrial networks (TN/NTN) including RSUs, UAVs, HAPs, and satellites. Future work will explore integrating DLaaS-inspired learning slices alongside our priority-based slices, enabling hybrid systems that support both mission-critical V2V communication and distributed learning tasks on the same infrastructure. Alongside learning-based methods, recent works investigated UAV-based data collection and optimized the Age of Information (AoI) as the optimization criterion. The authors in [39] studied the long-term average AoI minimization problem in UAV-assisted wireless-powered communication networks (WPCNs) by designing optimal transmission power of sensor nodes, optimal clustering of islands and UAV’s optimal flight trajectory. They suggested a hybrid TDMA and NOMA (HTN) protocol and a clustering-based dynamic adjustment of the shortest path (C-DASP) algorithm. They point out that the number of clusters and the total flying/hovering time are traded-off due to the battery capacity of the UAVs and, directly, on the performance of AoI.

## 3. System Model

The proposed architecture implement 5G NR for providing better QoS to support heterogeneous vehicular traffic. The simulation implements to mitigate the network congestion which consist of key components: 5G NR gNodeBs (gNBs), User Equipment (UE) which are vehicles (V), and Remote Host, a server application for generating traffic.

### 3.1. Vehicle Communication Model

The vehicle mobility model is implemented using a mobility model with instantaneous velocity S. Suppose the set of N connected vehicles in the simulation area is $V=\{v_{1},v_{2},\ldots ,v_{n}\}$ and the area is A = 500 × 500 m^2^. The positions of the vehicles are generated in accordance with the Random Waypoint mobility model: $UE_{i}\left(t\right)=\left(x_{i}\left(t\right),y_{i}\left(t\right),z_{i}\left(t\right)\right)$, Each vehicle v_*i*_ has a unique active application and it is pre-assigned to a unique slice s from the set A, B, C, D.

### 3.2. 5G NR Network

In this simulation, the set of gNBs denoted as BS are deployed using random allocator at a uniform height. The Constant Position Mobility Model, which denotes a fixed position for the gNB, serves as the gNB’s mobility model $gNB\left(t\right)=(x_{gNB}\left(t\right),y_{gNB}\left(t\right))$. The positions of BS are stationary in area:

(1) $$BS_{i}=\left[x_{i},y_{i},z_{i}\right]$$

where $z_{i}$ represent height of BS, which is constant.

The vehicles to base stations gNBs association is based on the principle of minimum Euclidean distance with respect to the gNB $BS_{i}$ to be served by *v:*

(2) $$BS_{i}\left(v,t\right)=\;{∥S_{v}\left(t\right)-S_{BS}∥}_{2}.$$

Implementing V2X applications requires a comprehensive approach that goes beyond improving connectivity in the radio access network (RAN). To meet these demands, the concept of network slicing emerges as a potential E2E solution that integrates the 5G system’s RAN and CN.

### 3.3. Network Slicing in the IoV

Network congestion in the IoV can be managed effectively through the use of network slicing. This enables different types of traffic to be isolated and managed separately, improving network efficiency and reducing congestion. From a security perspective, network slices are logically isolated from each other, preventing congestion or attacks in one slice from affecting others. In the context of the IoV, network slicing can ensure that critical vehicle-to-vehicle communication and navigation services receive the necessary bandwidth and low latency, even in high-density scenarios. This can help to ensure the reliable operation of the network and the safety of connected vehicles.

#### 3.3.1. Proposed Slices

**Figure 2:**
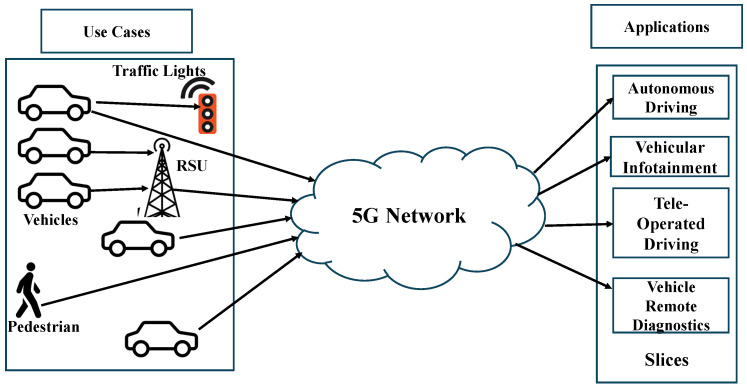
Generic View of Proposed Slices.

Based on the functional requirements of various IoV use cases, we propose the following network slices which are illustrated in Figure 2.

#### 3.3.2. Autonomous Driving (Slice A)

For self-driving cars, this slice provides highly reliable communication with extremely low latency. It facilitates the real-time exchange of vital safety information between vehicles (V2V). This guarantees that self-driving cars can avoid collisions and make decisions instantly.

#### 3.3.3. Vehicular Infotainment (Slice B)

This slice offers high-throughput communication for in-car entertainment and information services. It enables access to cloud-based content and smooth media streaming. High-quality music and video are available to passengers, improving their journey.

#### 3.3.4. Tele-Operated Driving (Slice C)

This slice delivers extremely low-latency and ultra-reliable connectivity between a remote operator and a vehicle. It is used in specific circumstances where a human operates the vehicle remotely. This guarantees accurate and safe control even when operating remotely.

#### 3.3.5. Vehicle Remote Diagnostics and Management (Slice D)

This slice supports the efficient exchange of small amounts of data for vehicle health monitoring and maintenance. It enables general fleet management, software updates, and remote diagnostics. This reduces vehicle downtime and supports proactive maintenance.

**Figure 3:**
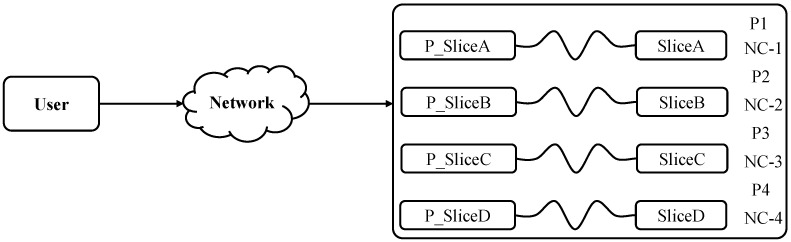
Port-Based Classification.

**Figure 4:**
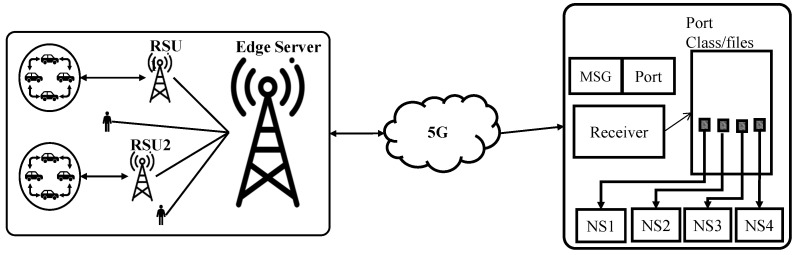
Traffic Routing to Specific Slice.

Traffic generated by connected vehicles is classified using a two-tier scheme: (1) Port-based mapping, $TT_{i}$ = Port$\left(S_{i}\right)$, assigns UDP port to slices, shown in Figure 3. (2) V2X message-type override using CAM/DENM header fields per ETSI EN 302 637-3: DENM [40] messages (type = 2, emergency) are always routed to Slice A, preventing misclassification of emergency traffic. While port-based classification is used for simplicity in this proof of concept, real-world IoV deployments would require more sophisticated methods. This two-tier scheme reliably separates beacons and emergency alerts. Traffic routing ensures that dedicated resource blocks are used for each slice as shown in Figure 4, which directly impacts performance. Port-based classification maps traffic to slices with <1 ms processing overhead. Routing decisions are made per-packet at the SDN controller. Slices are differentiated using the slice differentiator (SD). Each slice is identified by an S-NSSAI, which consists of two parts: The slice service type (SST) and the SD, which is 8 or 24 bits.

### 3.4. Network Slice Creation

Algorithm 1 is designed to create network slices for eMBB (slice B) and UrLLC (slice A, C, D) services. It begins by collecting service requirements and registering them with a Slicer/SDN. The algorithm then optimizes the parameters, such as eMBB bandwidth (BW) and each URLLC slice’s bandwidth, to meet the specified load requirements (LR) and traffic requirements (TR). If the QoS for eMBB or UrLLC is satisfied, the algorithm performs slice optimization; otherwise, it iterates through the optimization process until QoS is met. The computational complexity of this is O(N) where N is the number of slices. With N = 4, the algorithm executes at most 7 operations plus up to 5 QoS convergence iterations. Total runtime < 1 ms.
**Algorithm 1** Slice Creation for eMBB and UrLLC**Require:**  eMBB Parameters: ${\mathrm{BW}}_{\mathrm{eMBB}},{\mathrm{TP}}_{\mathrm{eMBB}},{\mathrm{LR}}_{\mathrm{eMBB}},{\mathrm{TR}}_{\mathrm{eMBB}}$  UrLLC Parameters: $N_{\mathrm{slices}},{\mathrm{BW}}_{\mathrm{UrLLC}}\left[\right],{\mathrm{TP}}_{\mathrm{UrLLC}}\left[\right],{\mathrm{LR}}_{\mathrm{UrLLC}}\left[\right],{\mathrm{TR}}_{\mathrm{UrLLC}}\left[\right]$1:**Step 1:** Collect service requirements2:**Step 2:** Register services with Slicer/SDN Controller3:**Step 3:** Slice creation for eMBB:4:   Optimize ${\mathrm{BW}}_{\mathrm{eMBB}},{\mathrm{TP}}_{\mathrm{eMBB}}$ to meet ${\mathrm{LR}}_{\mathrm{eMBB}},{\mathrm{TR}}_{\mathrm{eMBB}}$5:**if** QoS == eMBB satisfied **then**6:       Perform operation as slice optimization7:**else**8:       **Go to Step 3**9:**end if**10:**Step 4:** Slice creation for UrLLC:11:**for** i=1 **to** $N_{\mathrm{slices}}$ **do**12:       Optimize ${\mathrm{BW}}_{\mathrm{UrLLC}}\left[i\right],{\mathrm{TP}}_{\mathrm{UrLLC}}\left[i\right]$ to meet ${\mathrm{LR}}_{\mathrm{UrLLC}}\left[i\right],{\mathrm{TR}}_{\mathrm{UrLLC}}\left[i\right]$13:    **if** QoS == UrLLC satisfied **then**14:           Provide services according to slice optimization15:    **else**16:           **Go to Step 4**17:    **end if**18:**end for**19:**Step 5:** Return created slices20:**Step 6:** End

**Figure 5:**
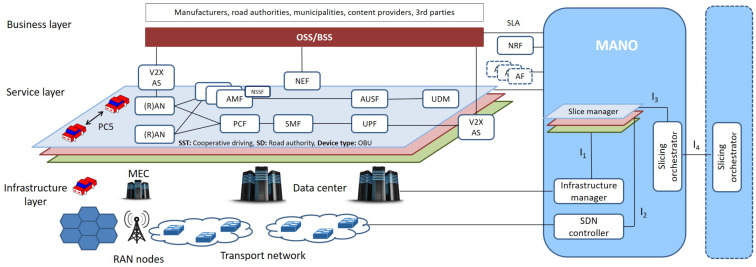
Network Slicing Framework [41].

Various slices can be created and managed in the context of the IoV defined in Algorithms 1 and 2. To simplify the management of slice roaming, we propose a standard set of essential functionalities that should be provided to a V2X slice, which can be agreed upon by all operators. These functionalities include the availability of AMF (Access and Mobility Management Function) instances to support vehicle mobility. Multiple dedicated AMF instances can be dynamically deployed to prevent overload during slice selection/attachment procedures and ensure isolation from other non-V2X slices. Additionally, NFs (Network Functions) such as AUSF (Authentication Server Function), PCF (Policy Control Function), and UDM (Unified Data Management) should be deployed as standard components for all V2X slices. These basic functionalities form the foundation of a default V2X slice. However, different functions can be dynamically chained and configured to meet the specific performance requirements of different V2X service categories. The slices can communicate V2N communications with the V2X Application Server (AS). High-reliability and low-latency transmission of raw sensory data from vehicles to the infrastructure, along with the exchange of computed results for extended spatial vehicle perception, are essential. Figure 5 illustrates the resulting NF chaining for the slices. The proposed framework aligns with ongoing 3GPP discussions on slice lifecycle operations. Regarding slice attachment, the framework follows the guidelines and customizes the procedure for connected vehicles. A Vehicle User Equipment (VUE) wishing to attach to a slice provides the Network Slice Selection Assistance Information (NSSAI) to the network, specifying the desired slice instance. For the V2X slice, the NSSAI parameters are set according to the slice’s specific requirements. The NSSF, located with the AMF, performs the slice selection procedure. Algorithm 2 is responsible for selecting the slice based on various parameters such as slice information (${\mathrm{Slice}}_{i}$), Number of connected nodes (${\mathrm{nodes}}_{n}$), Port identification, Delay (${\mathrm{delay}}_{Li}$), Quality of Service (${\mathrm{QoS}}_{\mathrm{class}}$), and available bandwidth (BW). Furthermore, the complexity of the algorithm with a constant time check is O(1) per vehicle. For V vehicles, total complexity is O(V) per control cycle.
**Algorithm 2** Slice Management**Require:**  Slice identifier: ${\mathrm{Slice}}_{i}$  Number of nodes: ${\mathrm{nodes}}_{n}$  Current delay: ${\mathrm{delay}}_{Li}$  QoS class: ${\mathrm{QoS}}_{\mathrm{class}}$  Bandwidth: BW**Ensure:**  Selected slice  ${\mathrm{Slice}}_{i},\;{\mathrm{consumer}}_{u},\;{\mathrm{delay}}_{Li},\;{\mathrm{QoS}}_{\mathrm{class}},\;\mathrm{BW}$1:**function** get_consumable_share2:    **if** ${\mathrm{nodes}}_{n}==0$ **then**3:        **return** $\min(m_{\mathrm{BW}})$           ▹ Minimum of max bandwidth constraints4:    **else**5:        **return** $\min\left(\frac{\mathrm{initial}\_\mathrm{cap}}{{\mathrm{nodes}}_{n}},m_{\mathrm{BW}}\right)$6:    **end if**7:**end function**8:**function** is_available9:    $\mathrm{real}\_\mathrm{cap}\leftarrow \min(\mathrm{(initial}\_\mathrm{cap},m_{\mathrm{BW}})$10:    $m_{\mathrm{BW}}=\sum_{i=1}^{N}{\mathrm{BW}}_{i}\leq \frac{{\mathrm{BW}}_{\mathrm{total}}}{{\mathrm{nodes}}_{N}+1}$               ▹ Bandwidth constraint11:    $\mathrm{actual}\_\mathrm{QoS}\leftarrow {\mathrm{delay}}_{Li}$              ▹ Current delay as QoS measure12:    **if** $\left({\mathrm{BW}}_{\mathrm{used}}<m_{\mathrm{BW}}\right)$ **and** $\left(\mathrm{actual}\_\mathrm{QoS}<{\mathrm{QoS}}_{\mathrm{class}}\right)$ **then**13:        **return True**14:    **else**15:        **return False**16:    **end if**17:**end function**

### 3.5. Resource Allocation

The network allocates dedicated resources (such as bandwidth) to each slice based on the requirements of different traffic types as specified in Algorithm 3. The priority weights $\alpha_{i}$ are dynamically adjusted every control cycle (100 ms) based on measured queue length $Q_{i}$ for each slice. The update rule is:

$$\alpha_{i}\left(t+1\right)=\alpha_{i}\left(t\right)\times \left(1+\beta \times \frac{Q_{i}^{\mathrm{target}}-Q_{i}}{Q_{i}^{\mathrm{target}}}\right)$$

where β=0.3 is the step size and $Q_{i}^{\mathrm{target}}$ is the target queue length for slice *i*.
**Algorithm 3** Bandwidth Allocation**Require:**  Total bandwidth: ${\mathrm{BW}}_{\mathrm{total}}$  Number of slices: *N*  Slice priority factors: $\alpha_{i}$ for i=1 to *N***Ensure:** Bandwidth allocation: ${\mathrm{BW}}_{i}$ for i=1 to *N*1:Initialize ${\mathrm{BW}}_{i}\leftarrow 0$ for all i=1 to *N*2:$\mathrm{total}\_\mathrm{alpha}\leftarrow \sum_{i=1}^{N}\alpha_{i}$            ▹ Calculate total weightage factor3:**for** i←1 **to** *N* **do**4:    ${\mathrm{BW}}_{i}\leftarrow \alpha_{i}\times \left({\mathrm{BW}}_{\mathrm{total}}/\mathrm{total}\_\mathrm{alpha}\right)$5:**end for**6:**if** $\sum_{i=1}^{N}{\mathrm{BW}}_{i}>{\mathrm{BW}}_{\mathrm{total}}$ **then**7:    $\mathrm{scaling}\_\mathrm{factor}\leftarrow {\mathrm{BW}}_{\mathrm{total}}/\sum_{i=1}^{N}{\mathrm{BW}}_{i}$8:    **for** i←1 **to** *N* **do**9:        ${\mathrm{BW}}_{i}\leftarrow {\mathrm{BW}}_{i}\times \mathrm{scaling}\_\mathrm{factor}$           ▹ Proportional adjustment10:    **end for**11:**end if**12:**return** ${\mathrm{BW}}_{i}$ for all *i*

Measured delay and packet loss are fed back to the SDN controller every Transmission Time Interval (TTI) of 1 ms. If ${\mathrm{delay}}_{i}>{\mathrm{delay}}_{i}^{\max}$, $\alpha_{i}$ is increased by 10% up to a maximum of 5× the base weight. Bandwidth reallocation occurs every 100 ms (control plane cycle), while per-packet routing decisions occur every TTI (1 ms).

Let N={A,B,C,D} be the slice set. The objective is to minimise the priority-weighted sum of end-to-end delays $J=\sum_{i\in N}w_{i}\cdot {\mathrm{delay}}_{i}\left(t\right)$ subject to bandwidth and QoS constraints:

(3) $$\begin{array}{ccc} \; & \left(C1\right)\;\sum_{i\in N}BW_{i}\leq BW_{\mathrm{total}}\; & [\mathrm{total}\;\mathrm{bandwidth}]\\ \; & \left(C2\right)\;BW_{i}\geq BW_{i}^{\min}\;\forall i\in N\; & [\mathrm{minimum}\;\mathrm{guarantee}\;\mathrm{per}\;\mathrm{slice}]\\ \; & \left(C3\right)\;{\mathrm{delay}}_{i}\leq {\mathrm{delay}}_{i}^{\max}\;\forall i\in N\; & [\mathrm{QoS}\;\mathrm{latency}\;\mathrm{constraint}]\\ \; & \left(C4\right)\;{\mathrm{PLR}}_{i}\leq {\mathrm{PLR}}_{i}^{\max}\;\forall i\in N\; & [\mathrm{packet}\;\mathrm{loss}\;\mathrm{constraint}]\\ \; & \left(C5\right)\;\alpha_{i}>0\;\forall i\in N,\;\alpha_{i}\in R^{+}\; & [\mathrm{positive}\;\mathrm{priority}\;\mathrm{weight}] \end{array}$$

where $w_{i}$ is the priority weight and $delay_{i}\left(t\right)$ is the current measured end-to-end delay for slice i, $BW_{i}^{min}$ is the minimum guaranteed bandwidth, and $PLR_{i}^{max}$ is the maximum tolerable packet loss ratio per slice.

Bandwidth is allocated proportionally using slice priority weights $\alpha_{i}$. The priority weights $\alpha_{i}$ are updated every 10 Hz control cycle based on measured queue length $Q_{i}$ for each slice. The resource allocation factor for slice *i* is:

$$F_{i}=\frac{\alpha_{i}}{\sum_{j\in N}\alpha_{j}}$$

$$BW_{i}=F_{i}\times BW_{\mathrm{total}}=\left(\frac{\alpha_{i}}{\sum \alpha_{j}}\right)\times BW_{\mathrm{total}}$$

Initially, bandwidth is allocated proportionally using weighted factors (α) that reflect each slice’s priority, ensuring higher allocation to critical slices; for instance, with a total bandwidth of 100 Mbps and weights $\alpha_{A}=4$, $\alpha_{B}=3$, $\alpha_{C}=2$, $\alpha_{D}=1$. As traffic conditions change, these weights are dynamically adjusted. For instance, if Slice A experiences higher demand, bandwidth is reallocated from lower-priority slices, ensuring critical services maintain their QoS while optimizing network performance. The bandwidth efficiently distributes the BW_total_$\sum {\mathrm{BW}}_{i}\in N$, ${\mathrm{BW}}_{i}\leq$ Total_Res, ∀i∈N among multiple network slices (*N*) based on factors (α) according to required resource of each *N*. It begins by initializing the bandwidth allocation BW_*i*_ = BW_total_× Total BW_*i*_ for each slice (BW_*i*_) to zero. Then, it calculates the total weightage factor ($\Sigma \alpha_{i}$) to ensure proportional allocation. The computational complexity is O(N) with 4N + 3 operations. For N = 4, this equals 19 operations per allocation cycle with runtime < 10 μs.

## 4. Simulation Framework

The simulation is implemented using the NS-3.37 simulator with a 5G-LENA module. The 500 × 500 m^2^ simulation region defines the geographic scope of the simulated network environment. There are 4 slices in the system, each with a different packet size. The simulation uses a packet size of 6144 bytes to reflect the size of each packet’s data transmission for infotainment traffic. However, other slices use standard bytes as defined by 3GPP. The allotted bandwidth for Band is set at 100 MHz, which affects the network’s capacity and performance. The simulation lasts 1000 ms, the value chosen for the simulation time. The subcarrier spacing and symbol time in the system are defined by the numerology value, set to 3, and a frequency of 28 GHz supporting broadcast and a bit rate of >7 Gbps. The signal processing technique is set to beamforming, which provides efficient signal strength, increases the signal-to-noise ratio (SNR), extends coverage, and reduces interference. It uses the UDP protocol. There are several numbers of nodes in the simulation, reflecting the different numbers of network entities. The nodes’ transmission power is set to 20 dBm, and the speed is 30 m/s.

### 4.1. Results Discussion

**Figure 6:**
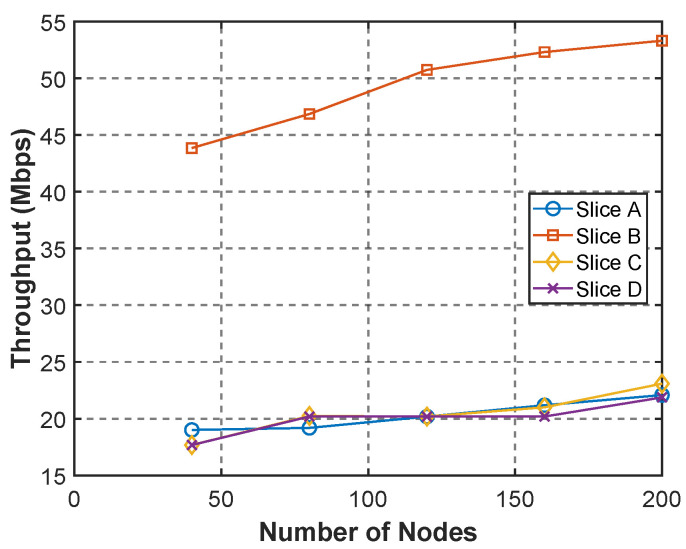
Throughput of Four Slices.

**Figure 7:**
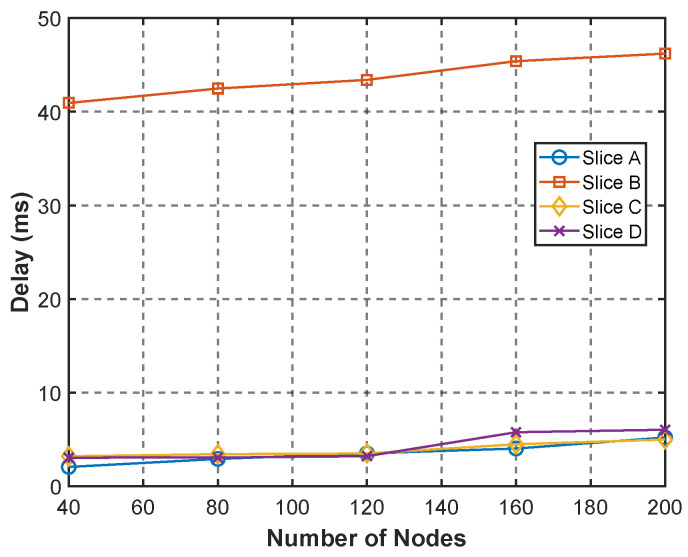
Delay of Four Slices.

**Figure 8:**
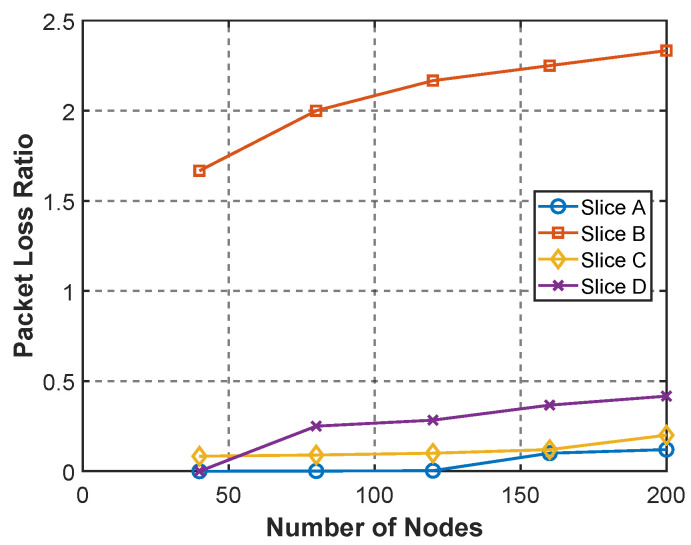
Packet Loss Ratio of Four Slices.

Four slices, A, B, C, and D, are simulated, and the results based on throughput (Figure 6), delay (Figure 7), and packet loss ratio (Figure 8) are discussed as

#### 4.1.1. Throughput vs. Node Density

Slice A exhibits relatively consistent throughput across the node counts in Figure 6. At 40 nodes, the throughput is 19.0088 units. At the highest node count of 200, the throughput reaches 22 Mbps. These results indicate that Slice A can maintain a stable and satisfactory level of data throughput for autonomous driving and safety-critical services.

Slice B shows a notable increase in throughput compared to the other slices because it is designed for high throughput. At 40 nodes, the throughput is 43.850667 units, which significantly exceeds that of the different slices. At 200 nodes, it reaches its maximum of 53 Mbps. These results indicate that Slice B can provide sufficient throughput for web browsing, social media access, app and file downloads, and HD video streaming for passengers in vehicular infotainment applications.

Slice C demonstrates lower throughput than Slices A and B. At 40 nodes, the throughput is 17.678293 units. It moderately increases and remains relatively stable at 23 units for 200 nodes. These results suggest that Slice C is highly capable of supporting fast vehicle control and feedback required for tele-operated driving scenarios.

Slice D exhibits throughput levels similar to Slice C. At all node counts, the throughput remains between 17.678293 and 21 units. This suggests that Slice D can effectively handle the exchange of low-frequency data between vehicles and remote servers for remote diagnostics and fleet management purposes.

#### 4.1.2. End-to-End Delay vs. Node Density

Slice A demonstrates relatively low delay values across all node counts in Figure 7. At 40 nodes, the delay is 2 ms. At the highest node count of 200, the delay reaches 5 ms. These results indicate that Slice A can maintain low-latency communication for time-critical safety applications in autonomous driving. Slice B exhibits significantly higher delay compared to the other slices. At 40 nodes, the delay is 40 ms, which is considerably higher. The delay remains consistently high at 200 nodes (46 ms). These results highlight the challenges in achieving ultra-low latency for vehicular infotainment application scenarios. Slice C demonstrates relatively low delay values similar to Slice A. At 40 nodes, the delay is 3 ms. At 200 nodes, the delay increases to 5 ms. Slice D exhibits consistently low delay values across all node counts. At all node counts, the delay remains below or around 3 ms, with the highest value at 6 ms at 200 nodes.

#### 4.1.3. Packet Loss Ratio (PLR) vs. Node Density

Figure 8 shows PLRs for four slices. At the maximum vehicle density of 200 nodes, the exact packet loss ratios are: Slice A = 0.12, Slice B = 2.33, Slice C = 0.20, and Slice D = 0.42. As the number of nodes increases, Slice B, which demands more bandwidth, experiences significantly higher packet losses compared to the other slices, especially at 200 nodes. In contrast, the other slices (A, C, and D) maintain relatively stable packet loss ratios, indicating that lower-priority slices are more affected by bandwidth demand, while higher-priority slices experience fewer losses despite increased saturation.

### 4.2. Comparison of Proposed Approach to State-of-the-Art Approaches

The performance of the proposed scheme is evaluated through simulation and benchmarked against the methods presented in [42,43]. In [43], the authors discuss network slicing as a method to enhance network service efficiency. This approach facilitates network offloading and improves overall network efficiency and user experience in various scenarios, such as video streaming and IoT devices. The authors propose 5G network slicing techniques that leverage blockchain, reinforcement learning, and a combination of both. In addition, the authors in [42] propose a distributed congestion control strategy for IoV that couples a Harmony Search (HS)-based global optimizer and a Throughput Evaluation Priority Adjustment Model (TEPAM). HS dynamically tunes each node’s transmission range and rate to minimize delay and jitter. At the same time, TEPAM prioritizes high-priority flows and adapts per-path congestion windows in real time via batch estimation on MPTCP paths.

For slice-to-slice throughput and delay analysis, the proposed scheme is benchmarked against [43]. In addition, for the evaluation of throughput, end-to-end delay, and packet loss ratio (PLR) against nodes density, the proposed scheme is compared with [42].

Given the comparable throughput and latency requirements, the slices considered in [43] are mapped to the corresponding slices of the proposed framework for performance comparison. Specifically, the Video Stream slice in [43] is compared with Slice B of the proposed work, the IoT Devices slice is compared with Slice A, and the Mobile Devices slice is compared with Slice C and D. The comparative analysis of these slices is presented below.

#### 4.2.1. Throughput Comparison

**Figure 9:**
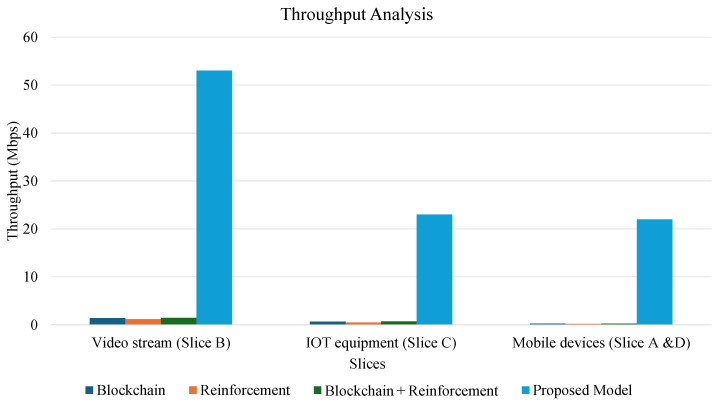
Comparison of Throughput to other techniques.

As shown in Figure 9, our proposed model’s throughput is evaluated against the blockchain, reinforcement learning, and hybrid variants of [43] for video streams (Slice B), IoT equipment (Slice C), and mobile devices (Slices A and D). For all evaluated slices, our proposed scheme consistently outperforms all the aforementioned methods, demonstrating superior resource allocation efficiency.

**Figure 10:**
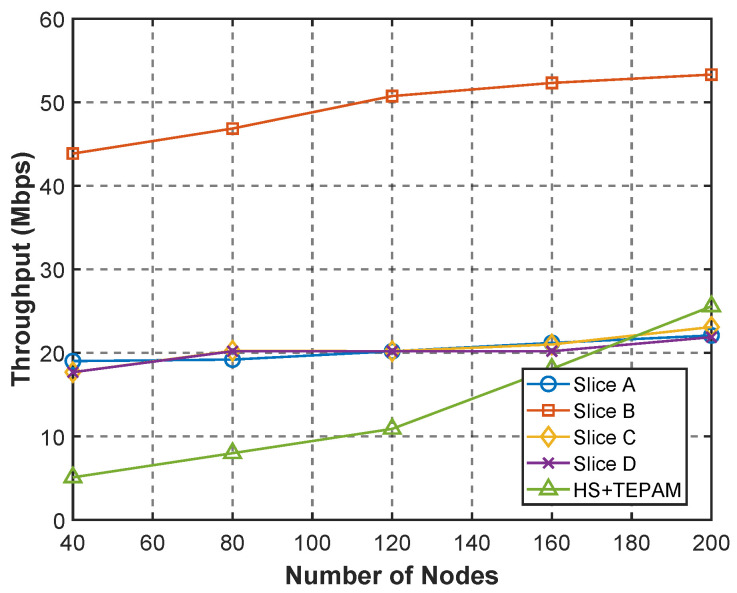
Throughput Comparison.

To evaluate the throughput performance in relation to node density, we compared the proposed method with the results from [42]. Figure 10 illustrates the throughput comparison. The HS + TEPAM method, which utilizes dynamic rate and range tuning, boosts throughput from approximately 5 Mbps with 40 vehicles to 25 Mbps with 200 vehicles. Our network-slicing approach offers four parallel slices: Slices C and D achieve modest increases from 17 Mbps to 23 Mbps, while Slice A maintains a throughput between 19 Mbps and 22 Mbps. Notably, Slice B, which is dedicated to high throughput, scales impressively to 40 Mbps.

#### 4.2.2. Delay Comparison

**Figure 11:**
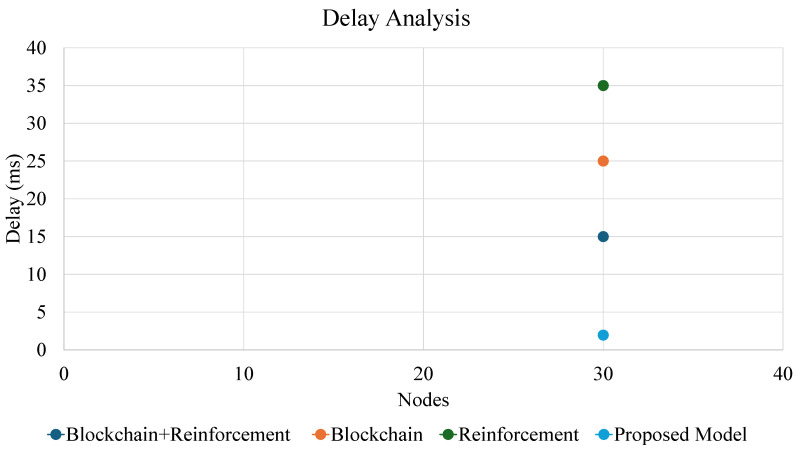
Comparison of Delay to Other Techniques.

As shown in Figure 11, our proposed model’s delay is evaluated against the blockchain, reinforcement learning, and hybrid variants of [43] using a fixed number of nodes. For this fixed number of nodes, our proposed scheme minimizes latency relative to the individual blockchain and reinforcement learning implementations in [43], demonstrating superior latency optimization.

**Figure 12:**
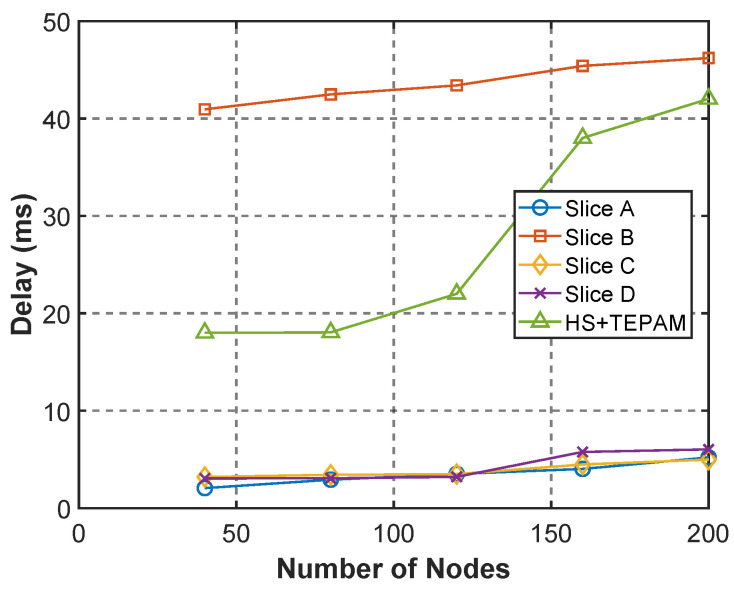
Delay Comparison.

To evaluate the delay performance in relation to node density, we compared the proposed method with the results from [42]. Figure 12 illustrates the average delay based on the number of vehicles. The authors in [42] optimized link parameters and window control together, reducing the delay from 42 ms to 18 ms. In comparison, our slices perform as follows: Slice A maintains an ultra-low delay of under 5 ms across all densities, Slice C stays below 10 ms, and Slice D remains below 6 ms. However, the delay performance for Slice B consistently degrades compared to the findings in [42]. This is primarily because Slice B is bandwidth-intensive and has a large packet size with continuous intervals, which leads to network congestion. Despite this, the high delays observed in Slice B do not impact the URLLC slices. 

#### 4.2.3. Packet Loss Ratio Comparison

**Figure 13:**
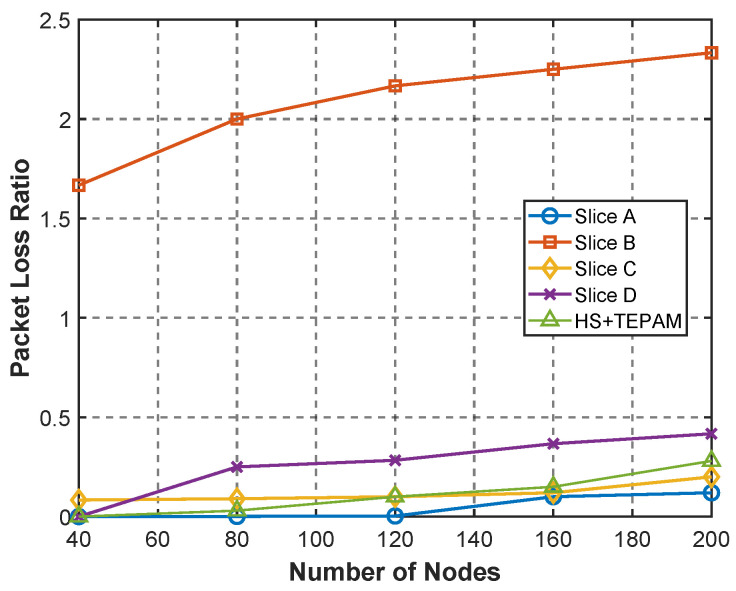
Packet Loss Ratio Comparison.

Figure 13 shows the PLR with an increasing number of nodes for the proposed scheme against [42]. The method presented in [42] increases the PLR from 0 at 40 vehicles to 0.35 at 200 vehicles. Under our proposed architecture, Slices A and C successfully maintain a strict PLR of less than 0.30 across all node densities, while Slice D shows an acceptable, slightly higher PLR of 0.45. Conversely, the high-bandwidth Slice B exhibits an increase in PLR from 1.6 to 2.3 under heavy load conditions. This behavior clearly demonstrates the efficacy of our slice isolation strategy, which ensures that critical traffic remains robustly protected even when high-bandwidth flows become vulnerable to congestion. All simulation results reported above are based on single-run simulations due to computational constraints. Future work will extend the analysis to n=30 independent seeds with full statistical reporting.

## 5. Conclusions

The IoV is a decentralized network designed to enable secure, seamless value transfer across various networks. However, an increase in network requests can lead to delays and network congestion, resulting in low throughput and high latency. To overcome this challenge, the IoV employs the concept of network slicing, which enables the development of virtual networks that utilize a single physical infrastructure. The proposed work focuses on a network-slicing-based strategy to mitigate congestion in IoV networks, enabling a congestion-free network with low latency, high throughput, and efficient resource use. The results show that the slicing approach provides better performance compared to the state of art and efficiently controls network congestion. The proposed approach is a stone toward the implementation of network slicing in Internet of Vehicles networks in the future to achieve improved performance, congestion control, and spectrum utilization in IoV environments. However, the current technique has some limitations, including reliance on simulation-based validation. Future research should concentrate on AI-powered adaptive slicing, real-world testbed deployment, and safe, energy-efficient frameworks. While real-world implementation of 5G SDN/NFV with MEC can ensure QoS and effective resource allocation, its practical deployment must address challenges such as infrastructure costs, interoperability, and seamless mobility management.

## Data Availability

The original contributions presented in this study are included in the article. Further inquiries can be directed to the corresponding author.
